# Differences between the oral changes presented by patients with solid and hematologic tumors during the chemotherapeutic treatment

**DOI:** 10.1590/1678-7757-2019-0020

**Published:** 2019-11-11

**Authors:** Isabella Lima Arrais Ribeiro, Sâmara Munique Silva, Rebecca Rhuanny Tolentino Limeira, Paulo Rogério Ferreti Bonan, Ana Maria Gondim Valença, Eufrásio Andrade de Lima, Ricardo Dias de Castro

**Affiliations:** 1 Universidade Federal da Paraíba, João Pessoa, Paraíba, Brazil; 2 Universidade Federal da Paraíba, Departamento de Clínica Social e Odontologia, João Pessoa, Paraíba, Brazil.; 3 Universidade Federal da Paraíba, Programa de Pós-Graduação em Decisão e Modelos de Saúde, João Pessoa, Paraíba, Brazil.

**Keywords:** Oral health, Oral manifestations, Antineoplastic agents, Cancer, Pediatrics

## Abstract

**Objective:**

This study sought to identify the differences between the oral changes presented by patients with solid and hematologic tumors during chemotherapeutic treatment.

**Methodology:**

This is an observational, prospective and quantitative study using direct documentation by follow-up of 105 patients from 0 to 18 years using the modified Oral Assessment Guide (OAG). Of the 105 patients analyzed, 57 (54.3%) were boys with 7.3 years (±5.2) mean age. Hematologic neoplasms accounted for 51.4% of all cases.

**Results:**

Voice, lips, tongue, and saliva changes were not significantly different (p>0.05) between patients with solid or hematologic tumors and during the follow-up. From the 6^th^ until the 10^th^ week of chemotherapeutic treatment alterations in swallowing function, in the mucous membrane (buccal mucosa and palate), in the labial mucosa, and in the gingiva occurred and were distributed differently between the two tumors groups (p<0.05). The main alterations were observed in patients with hematologic tumors.

**Conclusion:**

It was concluded that the oral changes during the chemotherapeutic treatment occurred especially in swallowing function, in the mucous membrane, in the labial mucosa and in the gingiva, and these alterations were found mainly in patients with hematologic tumors.

## Introduction

Cancer is one of the most significant public health problems, and its global incidence has increased by approximately 20.0% over the last decade, with a projected onset of 27 million new cases in 2030.^1^

Pediatric cancers are rare when compared with those affecting adults, accounting for 1% to 3% of the malignant tumors worldwide. Although these tumors usually have brief latency periods and are aggressive and fast growing, they respond well to antineoplastic therapies, with positive prognoses and likelihoods of cure, provided they are diagnosed early.^2^^–^^4^

Chemotherapy is involved in most treatments for pediatric cancers,^5^ and acts on most types of tumors affecting children and adolescents via chemical agents that affect cell growth and division processes.^6^ This effect causes changes throughout the gastrointestinal tract, given the cell renewal rate of cancer. Younger patients are more likely to be affected by chemotherapy in the oral cavity.^5^ The main alterations include oral mucositis, xerostomia, dysgeusia, and difficulties in swallowing saliva and food.^5^^,^^7^

Oral mucositis is one of the most common and important alterations in the lips and oral cavity, and when associated to xerostomia these are responsible for extremely debilitating conditions such as the painful inflammatory/ulcerative reaction of the oral mucosa, which stands out among these alterations. From these changes, complications in oral functions also occur and patients may become unable to feed or communicate. Moreover, oral mucositis can spread throughout the gastrointestinal tract,^8^^,^^9^ resulting in severe discomfort that can prevent these individuals from chewing, swallowing, and speaking.^10^^–^^13^

Such ulcerations resulting from chemotherapy can cause intense pain and may require the use of opioid analgesics, hospitalization and complementary nutrition. Furthermore, oral mucositis may contribute to the interruption of cancer treatment, promoting survival reduction. The presence of these ulcers in immunocompromised patients may facilitate the entry of microorganisms into the body, increasing the risk of death from sepsis. This clinical condition thus represents a costly situation for public or private health systems.^14^

Extensive knowledge about the development of oral mucositis induced by chemotherapy in different clinical and pathological conditions is required for its prevention, control, and treatment. Different types of tumors require different therapeutic protocols, and this may represent a risk factor for the occurrence of lesions resulting from the direct action of chemotherapeutic agents on the mucosal epithelial cells or from the suppression of proinflammatory chemical mediators, for example of tumor necrosis factor (TNF-α), interleukin 1-β (IL-1β) and interleukin-6 (IL-6) produced by neutrophils and macrophages in response to the microorganisms present in the lesion.^15^ The presence of these cytokines in the saliva of cancer patients undergoing treatment is also significantly associated with oral candidosis lesions and HSV infection.^16^

The tumor type is one of the factors that affect the occurrence, severity and duration of oral mucositis.^8^^,^^17^ Therefore, a differentiated approach for pediatric cancer patients with hematologic and solid tumors is crucial, given that they may present different oral changes during chemotherapy. Given this context, this study sought to identify the major oral damage related to chemotherapy among pediatric cancer patients, and to evaluate the differences between patients with solid and hematologic tumors.

## Methodology

### Study design

This is an observational, longitudinal, prospective, and quantitative study using an inductive approach, and a comparative-statistical procedure by a direct documentation technique.

### Ethical considerations

This study was approved by the Human Research Ethics Committee under Certificate of Presentation for Ethical Appreciation no. 12922113.8.0000.5188.

### Patients

The patients evaluated were between 0 and 18 years old and treated at the Hospital Napoleão Laureano located in João Pessoa, Paraíba, Brazil. This hospital is recognized as a reference center for the prevention, diagnosis, and treatment of cancer in the state of Paraíba, performing approximately 7,000 monthly patient visits, including consultations, examinations and surgeries, and providing treatment to 3,300 patients *per* month on average.

Inclusion criteria – Patients between 0 and 19 years old with a primary diagnosis of malignant neoplasm treated at the Hospital by the Brazilian Unified Health System (SUS) with chemotherapy as the exclusive treatment over the first months were considered as eligible for the current study. In oncology, the ages between 0 to 19 years old are considered as the pediatric age group since the tumors in children and adolescents within this age range behave in a similar way regarding the development and response to treatment; thus, being treated by pediatric oncologists.^2^ For this reason, the inclusion criteria of this study established the minimum age for inclusion of 0 years old and maximum of 19 years old.

Exclusion criteria – Patients with oral mucosa inflammation at diagnosis, initiation of radiotherapy or surgical treatment concomitant with chemotherapy before completing the evaluation period of the study were excluded from this study. Furthermore, patients with re-initiation of treatment for recurrent neoplasm, impaired health status, and interruption (for any reason) of the follow-up of this study were also excluded.

### Sample

The hospital census sample was recruited between April 2013 and July 2015, for 105 patients in total.

### Data collection

Data collection was performed at the dental office of the Pediatrics Department and at the bedsides of inpatients. The evaluations were performed by a single and previously calibrated examiner (kappa=0.87), using artificial lighting to improve the visualization of the oral cavity.

**Figure 1:**
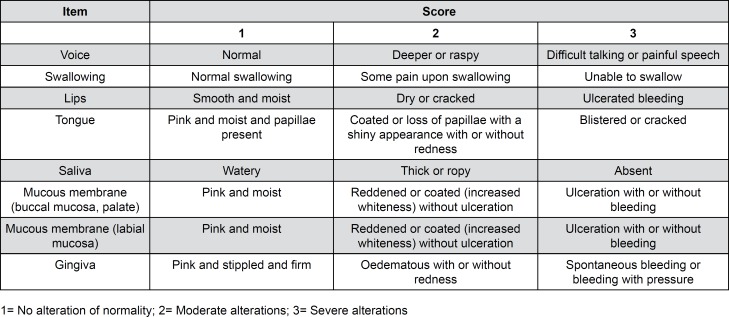
Modified Oral Assessment Guide to monitor the oral health of patients undergoing chemotherapy

The modified Oral Assessment Guide (OAG; Figure 1) was used for data collection. This guide evaluates the oral functions and structures according the degree of commitment, and is recognized by the scientific community for the evaluation of changes in the oral mucosa resulting from antineoplastic treatment using chemotherapeutic agents. This instrument evaluates 8 items according to the oral health impairment scale, scoring each item from 1 to 3, being: 1=normal conditions; 2=mild-to-moderate changes in epithelial integrity or function; and 3=severe impairment, with severe alterations to epithelial integrity or function.^17^^–^^20^

The oral health conditions were monitored for 10 weeks from the start of chemotherapy because this period is critical for the onset of oral alterations resulting from chemotherapy.^19^ The chemotherapeutic agent classes that were administered to patients in each week of treatment were also collected.

### Data analysis

Descriptive and inferential statistics (association tests such as the Chi-square test with Yates's continuity correction) were applied to analyze the data using IBM SPSS 21.0 at 5% significance level.

## Results

The mean age of the patients was 7.3 (±5.2) years, with a median of 7.3 years (range=0-18 years), with a higher concentration of malignant neoplasms at the ages of 2 (n=18; 17.0%), 3 (n=10; 9.5%), and 4 years (n=16; 15.2%), as well as among boys (n=57; 54.3%).

Of the patients evaluated, 48.6% (n=51) were diagnosed with solid tumors primarily located in the left kidney (Wilms's tumor; n=13; 25.5%), followed by the right femur (osteosarcoma; n=9; 17.6%). Of the patients with hematologic tumors, the most prevalent underlying disease was acute lymphoblastic leukemia (n=42; 77.7%).

Tables 1, 2, and 3 present the distribution of pediatric cancer patients according to the degree of changes in the structures/functions of the stomatognathic system over the 10-week evaluation after starting chemotherapy. No significant differences (p>0.05) were found between patients with solid tumors and those with hematologic tumors with regard to the changes assessed in the OAG (i.e., voice, lips, tongue, and saliva) until the 5^th^ week of chemotherapeutic treatment.

**Table 1:** Distribution of pediatric cancer patients by the structural impairment degree of the stomatognathic system in the first 4 weeks of evaluation following chemotherapy onset

			None	Moderate	Severe	Total	
**1**	Voice	1	49 (96.1%)	2 (3.9%)	-	51 (100.0%)	1.000
		2	51 (94.4%)	3 (5.6%)	-	54 (100.0%)	
	Swallowing	1	47 (92.2%)	4 (7.8%)	0 (0.0%)	51 (100.0%)	0.428
		2	51 (94.4%)	2 (3.7%)	1 (1.9%)	54 (100.0%)	
	Lips	1	33 (64.7%)	13 (25.5%)	5 (9.8%)	51 (100.0%)	0.743
		2	35 (64.8%)	16 (29.6%)	3 (5.6%)	54 (100.0%)	
	Tongue	1	47 (92.2%)	0 (0.0%)	4 (7.8%)	51(100.0%)	0.428
		2	51 (94.4%)	1 (1.9%)	2 (3.7%)	54 (100.0%)	
	Saliva	1	14 (27.5%)	31 (60.8%)	6(11.8%)	51 (100.0%)	0.764
		2	16 (29.6%)	34 (63.0%)	4 (7.4%)	54 (100.0%)	
	Mucous membrane (buccal mucosa, palate)	1	48 (94.1%)	2 (3.9%)	1 (2.0%)	51 (100.0%)	0.111
		2	54 (100.0%)	0 (0.0%)	0 (0.0%)	54 (100.0%)	
	Labial mucosa	1	49 (96.1%)	2 (3.9%)	-	51 (100.0%)	0.234
		2	54 (100.0%)	0 (0.0%)	-	54 (100.0%)	
	Gingiva	1	50 (98.0%)	0 (0.0%)	1 (2.0%)	51 (100.0%)	0.243
		2	50 (92.6%)	3 (5.6%)	1 (1.9%)	54 (100.0%)	
**2**	Voice	1	49 (96.1%)	2 (3.9%)	-	51 (100.0%)	1.000
		2	51 (94.4%)	3 (5.6%)	-	54 (100.0%)	
	Swallowing	1	50 (98.0%)	1 (2.0%)	-	51 (100.0%)	1.000
		2	53 (98.1%)	1 (1.9%)	-	54 (100.0%)	
	Lips	1	32 (62.7%)	10 (19.6%)	9 (17.6%)	51 (100.0%)	0.098
		2	26 (48.1%)	21 (38.9%)	7 (13.0%)	54 (100.0%)	
	Tongue	1	50 (98.0%)	-	1 (2.0%)	51 (100.0%)	1.000
		2	52 (96.3%)	-	2 (3.7%)	54 (100.0%)	
	Saliva	1	12 (23.5%)	34 (66.7%)	5 (9.8%)	51 (100.0%)	0.913
		2	12 (22.2%)	35 (64.8%)	7(13.0%)	54 (100.0%)	
	Mucous membrane (buccal mucosa, palate)	1	48 (94.1%)	3 (5.9%)	0 (0.0%)	51 (100.0%)	0.299
		2	51 (94.4%)	1 (1.9%)	2 (3.7%)	54 (100.0%)	
	Labial mucosa	1	40 (78.4%)	7(13.7%)	4 (7.8%)	51 (100.0%)	0.536
		2	40 (74.1%)	6(11.1%)	8 (14.8%)	54 (100.0%)	
	Gingiva	1	49 (96.1%)	1 (2.0%)	1 (2.0%)	51 (100.0%)	0.485
		2	51 (94.4%)	3 (5.6%)	0 (0.0%)	54 (100.0%)	
**3**	Voice	1	49 (96.1%)	-	2 (3.9%)	51 (100.0%)	0.234
		2	54 (100%)	-	0 (0.0%)	54 (100.0%)	
	Swallowing	1	47 (92.2%)	2 (3.9%)	2 (3.9%)	51 (100.0%)	0.327
		2	53 (98.1%)	1 (1.9%)	0 (0.0%)	54 (100.0%)	
	Lips	1	36 (70.6%)	8 (15.7%)	7 (13.7%)	51 (100.0%)	0.505
		2	33 (61.1%)	9 (16.7%)	12 (22.2%)	54 (100.0%)	
	Tongue	1	48 (94.1%)	1 (2.0%)	2 (3.9%)	51 (100.0%)	0.803
		2	52 (96.3%)	1 (1.9%)	1 (1.9%)	54 (100.0%)	
	Saliva	1	19 (37.3%)	27 (52.9%)	5 (9.8%)	51 (100.0%)	0.174
		2	12 (22.2%)	38 (70.4%)	4 (7.4%)	54 (100.0%)	
	Mucous membrane (buccal mucosa, palate)	1	47 (92.2%)	2 (3.9%)	2 (3.9%)	51 (100.0%)	0.165
		2	48 (88.9%)	0 (0.0%)	6 (11.1%)	54 (100.0%)	
	Labial mucosa	1	46 (90.2%)	2 (3.9%)	3 (5.9%)	51 (100.0%)	0.228
		2	43 (79.6%)	2 (3.7%)	9 (16.7%)	54 (100.0%)	
	Gingiva	1	47 (92.2%)	1 (2.0%)	3 (5.9%)	51 (100.0%)	0.322
		2	49 (90.7%)	4 (7.4%)	1 (1.9%)	54 (100.0%)	
**4**	Voice	1	48 (94.1%)	1 (2.0%)	2 (3.9%)	51 (10.00%)	0.850
		2	51 (94.4%)	2 (3.7%)	1 (1.9%)	54 (100.0%)	
	Swallowing	1	49 (96.1%)	1 (2.0%)	1 (2.0%)	51 (100.0%)	0.807
		2	50 (92.6%)	3 (5.6%)	1 (1.9%)	54 (100.0%)	
	Lips	1	36 (70.6%)	7 (13.7%)	8(15.7%)	51 (100.0%)	0.955
		2	36 (66.7%)	8 (14.8%)	10(18.5%)	54 (100.0%)	
	Tongue	1	46 (90.2%)	2 (3.9%)	3 (5.9%)	51(100.0%)	0.202
		2	51 (94.4%)	3 (5.6%)	0 (0.0%)	54 (100.0%)	
	Saliva	1	10 (19.6%)	36 (70.6%)	5 (9.8%)	51 (100.0%)	0.095
		2	16 (29.6%)	27 (50.0%)	11(20.4%)	54 (100.0%)	
	Mucous membrane (buccal mucosa, palate)	1	45 (88.2%)	5 (9.8%)	1 (2.0%)	51 (100.0%)	0.520
		2	48 (88.9%)	3 (5.6%)	3 (5.6%)	54 (100.0%)	
	Labial mucosa	1	44 (86.3%)	5 (9.8%)	2 (3.9%)	51 (100.0%)	0.575
		2	47 (87.0%)	3 (5.6%)	4 (7.4%)	54 (100.0%)	
	Gingiva	1	45 (88.2%)	3 (5.9%)	3 (5.9%)	51 (100.0%)	0.093
		2	53 (98.1%)	1 (1.9%)	0 (0.0%)	54 (100.0%)	

Chi-square test with Yates's continuity correction, α=5%

1= Solid tumors; 2= Hematologic tumors. Empty spaces= no record of degree of impairment according to the OAG regarding the anatomical site/function for the evaluation week

**Table 2:** Distribution of pediatric cancer patients by the structural impairment degree of the stomatognathic system of the 5^th^ to 8^th^ weeks of evaluation following chemotherapy onset

Week	Anatomical site//Function	Tumor type	Changes according to OAG category			Total	Sig.
			None	Moderate	Severe		
5	Voice	1	48 (94.1%)	2 (3.9%)	1 (2.0%)	51 (100.0%)	0.480
		2	53 (98.1%)	0 (0.0%)	1 (1.9%)	54 (100.0%)	
	Swallowing	1	49 (96.1%)	-	2 (3.9%)	51 (100.0%)	0.611
		2	53 (98.1%)	-	1 (1.9%)	54 (100.0%)	
	Lips	1	47 (92.2%)	4 (7.8%)	0 (0.0%)	51 (100.0%)	0.197
		2	52 (96.3%)	1 (1.9%)	1 (1.9%)	54 (100.0%)	
	Tongue	1	47 (92.2%)	4 (7.8%)	0 (0.0%)	51(100.0%)	0.197
		2	52 (96.3%)	1 (1.9%)	1(1.9%)	54 (100.0%)	
	Saliva	1	13 (25.5%)	33 (64.7%)	5 (9.8%)	51 (100.0%)	0.282
		2	20 (37.0%)	32 (59.3%)	2 (3.7%)	54 (100.0%)	
	Mucous membrane (buccal mucosa, palate)	1	50 (98.0%)	0 (0.0%)	1 (2.0%)	51 (100.0%)	0.118
		2	48 (88.9%)	4 (7.4%)	2 (3.7%)	54 (100.0%)	
	Labial mucosa	1	50 (98.0%)	0 (0.0%)	1 (2.0%)	51 (100.0%)	0.091
		2	47 (87.0%)	2 (3.7%)	5 (9.3%)	54 (100.0%)	
	Gingiva	1	49 (96.1%)	1 (2.0%)	1 (2.0%)	51 (100.0%)	0.807
		2	50 (92.6%)	3 (5.6%)	1 (1.9%)	54 (100.0%)	
6	Voice	1	50 (98.0%)	0 (0.0%)	1 (2.0%)	51 (100.0%)	0.738
		2	53 (98.1%)	1 (1.9%)	0 (0.0%)	54 (100.0%)	
	Swallowing	1	49 (96.1%)	0 (0.0%)	2 (3.9%)	51 (100.0%)	0.180
		2	52 (96.3%)	2 (3.7%)	0 (0%0)	54 (100.0%)	
	Lips	1	38 (74.5%)	6 (11.8%)	7 (13.7%)	51 (100.0%)	0.347
		2	34 (63.0%)	12 (22.2%)	8 (14.8%)	54 (100.0%)	
	Tongue	1	46 (90.2%)	1 (2.0%)	4 (7.8%)	51 (100.0%)	0.068
		2	52 (96.3%)	2 (3.7%)	0 (0.0%)	54 (100.0%)	
	Saliva	1	17 (33.3%)	29 (56.9%)	5 (9.8%)	51 (100.0%)	0.602
		2	16 (29.6%)	29 (53.7%)	9(16.7%)	54 (100.0%)	
	Mucous membrane (buccal mucosa, palate)	1	45 (88.2%)	6(11.8%)	-	51 (100.0%)	0.056
		2	53 (98.1%)	1 (1.9%)	-	54 (100.0%)	
	Labial mucosa	1	43 (84.3%)	7(13.7%)	1 (2.0%)	51 (100.0%)	0.001
		2	42 (77.8%)	1 (1.9%)	11 (20.4%)	54 (100.0%)	
	Gingiva	1	50 (98.0%)	0 (0.0%)	1 (2.0%)	51 (100.0%)	0.044
		2	47 (87.0%)	5 (9.3%)	2 (3.7%)	54 (100.0%)	
7	Voice	1	50 (98.0%)	-	1 (2.0%)	51 (100.0%)	0.486
		2	54 (100.0%)	-	0 (0.0%)	54 (100.0%)	
	Swallowing	1	49 (96.1%)	-	2 (3.9%)	51 (100.0%)	0.234
		2	54 (100.0%)	-	0 (0.0%)	54 (100.0%)	
	Lips	1	45 (88.2%)	2 (3.9%)	4 (7.8%)	51 (100.0%)	0.088
		2	53 (98.1%)	0 (0.0%)	1 (1.9%)	54 (100.0%)	
	Tongue	1	45 (88.2%)	2 (3.9%)	4 (7.8%)	51 (100.0%)	0.088
		2	53 (98.1%)	0 (0.0%)	1 (1.9%)	54 (100.0%)	
	Saliva	1	12 (23.5%)	34 (66.7%)	5 (9.8%)	51 (100.0%)	0.784
		2	16 (29.6%)	34 (63.0%)	4 (7.4%)	54 (100.0%)	
	Mucous membrane (buccal mucosa, palate)	1	51(100.0%)	0 (0.0%)	0 (0.0%)	51 (100.0%)	0.496
		2	51 (94.4%)	2 (3.7%)	1 (1.9%)	54 (100.0%)	
	Labial mucosa	1	47 (92.2%)	1 (2.0%)	3 (5.9%)	51 (100.0%)	0.852
		2	50 (92.6%)	0 (0.0%)	4 (7.4%)	54 (100.0%)	
	Gingiva	1	49 (96.1%)	1 (2.0%)	1 (2.0%)	51 (100.0%)	0.807
		2	50 (92.6%)	3 (5.6%)	1 (1.9%)	54 (100.0%)	
8	Voice	1	50 (98.0%)	-	1 (2.0%)	51 (100.0%)	0.486
		2	54 (100.0%)	-	0 (0.0%)	54 (100.0%)	
	Swallowing	1	46 (90.2%)	1 (2.0%)	4 (7.8%)	51 (100.0%)	0.071
		2	50 (92.6%)	4 (7.4%)	0 (0.0%)	54 (100.0%)	
	Lips	1	47 (92.2%)	2 (3.9%)	2 (3.9%)	51 (100.0%)	0.377
		2	50 (92.6%)	4 (7.4%)	0 (0.0%)	54 (100.0%)	
	Tongue	1	47 (92.2%)	2 (3.9%)	2 (3.9%)	51 (100.0%)	0.377
		2	50 (92.6%)	4 (7.4%)	0 (0.0%)	54 (100.0%)	
	Saliva	1	49 (96.1%)	0 (0.0%)	2 (3.9%)	51 (100.0%)	0.059
		2	50 (92.6%)	4 (7.4%)	0 (0.0%)	54 (100.0%)	
	Mucous membrane (buccal mucosa, palate)	1	49 (96.1%)	0 (0.0%)	2 (3.9%)	51 (100.0%)	0.059
		2	50 (92.6%)	4 (7.4%)	0 (0.0%)	54 (100.0%)	
	Labial mucosa	1	50 (98.0%)	0 (0.0%)	1 (2.0%)	51 (100.0%)	0.030
		2	45 (83.3%)	3 (5.6%)	6 (11.1%)	54 (100.0%)	
	Gingiva	1	50 (98.0%)	1 (2.0%)	0 (0.0%)	51 (100.0%)	0.427
		2	49 (90.7%)	2 (3.7%)	3 (5.6%)	54 (100.0%)	

Chi-square test with Yates's continuity correction, α=5

1= Solid tumors; 2= Hematologic tumors.

Empty spaces= no record of degree of impairment according to the OAG regarding the anatomical site/function for the evaluation week

**Table 3:** Distribution of pediatric cancer patients by the structural impairment degree of the stomatognathic system in the 9^th^ and 10^th^ weeks of evaluation following chemotherapy onset

Week	Anatomical site//Function	Tumor type	Changes according to OAG category			Total	Sig.
			None	Moderate	Severe		
9	Voice	1	50 (98.0%)	-	1 (2.0%)	51 (100.0%)	0.486
		2	54 (100.0%)	-	0 (0.0%)	54 (100.0%)	
	Swallowing	1	49 (96.1%)	0 (0.0%)	2 (3.9%)	51 (100.0%)	0.059
		2	50 (92.6%)	4 (7.4%)	0 (0.0%)	54 (100.0%)	
	Lips	1	46 (90.2%)	5 (9.8%)	0 (0.0%)	51 (100.0%)	1.000
		2	48 (88.9%)	5 (9.3%)	1 (1.9%)	54 (100.0%)	
	Tongue	1	46 (90.2%)	5 (9.8%)	0 (0.0%)	51 (100.0%)	1.000
		2	48 (88.9%)	5 (9.3%)	1 (1.9%)	54 (100.0%)	
	Saliva	1	12 (23.5%)	34 (66.7%)	5 (9.8%)	51 (100.0%)	0.543
		2	10 (18.5%)	41 (75.9%)	3 (5.6%)	54 (100.0%)	
	Mucous membrane (buccal mucosa, palate)	1	51(100.0%)	0 (0.0%)	-	51 (100.0%)	0.027
		2	48 (88.9%)	6 (11.1%)	-	54 (100.0%)	
	Labial mucosa	1	50 (98.0%)	0 (0.0%)	1 (2.0%)	51 (100.0%)	0.084
		2	47 (87.0%)	1 (1.9%)	6 (11.1%)	54 (100.0%)	
	Gingiva	1	51 (100.0%)	0 (0.0%)	-	51 (100.0%)	0.496
		2	52 (96.3%)	2 (3.7%)	-	54 (100.0%)	
10	Voice	1	50 (98.0%)	-	1 (2.0%)	51 (100.0%)	0.486
		2	54 (100.0%)	-	0 (0.0%)	54 (100.0%)	
	Swallowing	1	49 (96.1%)	0 (0.0%)	2 (3.9%)	51 (100.0%)	0.028
		2	49 (90.7%)	5 (9.3%)	0 (0.0%)	54 (100.0%)	
	Lips	1	51 (100.0%)	0 (0.0%)	0 (0.0%)	51 (100.0%)	0.085
		2	49 (90.7%)	4 (7.4%)	1 (1.9%)	54 (100.0%)	
	Tongue	1	51 (100.0%)	0 (0.0%)	0 (0.0%)	51(100.0%)	0.085
		2	49 (90.7%)	4 (7.4%)	1 (1.9%)	54 (100.0%)	
	Saliva	1	19 (37.3%)	27 (52.9%)	5 (9.8%)	51 (100.0%)	0.726
		2	17 (31.5%)	33 (61.1%)	4 (7.4%)	54 (100.0%)	
	Mucous membrane (buccal mucosa, palate)	1	51 (100.0%)	0 (0.0%)	-	51 (100.0%)	0.118
		2	50 (92.6%)	4 (7.4%)	-	54 (100.0%)	
	Labial mucosa	1	51 (100.0%)	0 (0.0%)	0 (0.0%)	51 (100.0%)	0.000
		2	42 (77.8%)	8 (14.8%)	4 (7.4%)	54 (100.0%)	
	Gingiva	1	51 (100.0%)	0 (0.0%)	0 (0.0%)	51 (100.0%)	0.040
		2	48 (88.9%)	1 (1.9%)	5 (9.3%)	54 (100.0%)	

Chi-square test with Yates's continuity correction, α=5%

1= Solid tumors; 2= Hematologic tumors.

Empty spaces= no record of degree of impairment according to the OAG regarding the anatomical site/function for the evaluation week

Conversely, from the 6^th^ week of treatment onwards, the oral changes began to appear differently between the two groups of patients with different types of tumors. For the better presentation of these results, the differences verified will be described in the sequence these alterations were evaluated by the data collection instrument (OAG).

Regarding swallowing function, a significant difference (p=0.028) in impairment was assessed between the patients with solid and hematologic tumors on the 10^th^ week, with 3.9% of patients with solid tumors showing moderate impairments (e.g., swallowing difficulty) when compared with 9.3% of patients with hematologic tumors showing the more severe alteration (e.g., swallowing impossibility).

In mucous membrane (buccal mucosa and palate), significant differences (p=0.027) were observed between patients with solid and hematologic tumors on the 9^th^ week, with no change in normality for patients with solid tumors when compared to those with hematologic tumors, with 11.1% of patients in this group showing moderate changes during this period.

Significant differences in the labial mucosa were found between patients with solid and hematologic tumors on the 6^th^ (p=0.001), 8^th^ (p=0.030), and 10^th^ (p<0.001) weeks. On the 6^th^ week, moderate impairment affected 11.8% more solid tumors patients than hematologic tumors patients. Severe alterations for the same site affected more the hematologic tumors patients (18.4% more than in solid tumors patients). On the 8^th^ week, the labial mucosa changes occurred mainly in hematologic tumors patients, considering the occurrence of moderate and severe alterations, respectively, 5.6% and 9.1% higher than in the solid tumors group. In the 10^th^ week, patients with solid tumors showed no changes in the normality of the labial mucosa when compared to 14.8% with moderate changes, and 7.4% with severe changes in the hematologic tumors group.

Significant differences in gingiva changes were found in patients with solid and hematologic tumors in the 6^th^ week (p=0.044), with 2.0% of the patients with solid tumors showing severe impairment (e.g., spontaneous bleeding), when compared to 3.7% of patients with hematologic tumors showing the same condition, and only hematologic tumors patients presented moderate alterations in gingiva (9.3%). On the 10^th^ week, a significant difference (p=0.040) was also found between the patient groups. Patients with solid tumors showed no changes, whereas 9.3% of the patients with hematologic tumors presented spontaneous gingiva bleeding.

Table 4 shows the distribution of the chemotherapeutic agent classes that were administered to patients with hematologic or solid tumors in each week of treatment. Except in the 3^rd^ week the antimetabolites agents – class that includes methotrexate and the 5-fluorouracil –, were administered mainly to patients with hematologic cancer, being the frequencies of: 1^st^ week (78.0%); 2^nd^ week (80.4%); 4^th^ week (79.6% when isolated and 100.0% in association to natural products); 5^th^ week (79.6%); 6^th^ week (81.2% when isolated and 100.0% in association to alkylating agents, natural and miscellaneous products); 7^th^ week (81.1% when isolated and 100.0% in association to alkylating agents, natural and miscellaneous products); 8^th^ week (82.1% when isolated and 100.0% in association to alkylating agents, natural and miscellaneous products); 9^th^ week (82.0% when isolated and 100.0% in association to alkylating agents, natural and miscellaneous products); and in the 10^th^ week (81.1%).

**Table 4:** Distribution of patients with solid and hematological tumors according to the chemotherapy protocol used in the treatment of the neoplasia, in each of the weeks of the study follow-up

Week	Chemotherapeutic treatment	Group of patients		Sig.
		Solid tumors	Hematologic tumors	
1^st^	1	13 (72.2%)	5 (27.8%)	<0.001
	2	9 (22.0%)	33 (78.0%)	
	3	25 (65.8%)	13 (34.2%)	
	4	1 (25.0%)	3 (75.0%)	
2^nd^	1	10 (90.9%)	1 (9.1%)	<0.001
	2	9 (19.6%)	37 (80.4%)	
	3	28 (70.0%)	12 (30.0%)	
	4	1 (25.0%)	3 (75.0%)	
	Association (1, 3 and 4)	3 (100.0%)	0 (0.0%)	
	Association (2 and 3)	0 (0.0%)	1 (100.0%)	
3^rd^	1	8 (66.7%)	4 (33.3%)	<0.001
	2	10 (100.0%)	0 (0.0%)	
	3	30 (68.2%)	14 (31.8%)	
	4	0 (0.0%)	3 (100.0%)	
	Association (1, 3 and 4)	3 (100.0%)	0 (0.0%)	
4^th^	1	6 (75.0%)	2 (25.0%)	<0.001
	2	10 (20.4%)	39 (79.6%)	
	3	32 (78.0%)	9 (22.0%)	
	4	0 (0.0%)	3 (100.0%)	
	Association (1, 3 and 4)	3 (100.0%)	0 (0.0%)	
	Association (2 and 3)	0 (0.0%)	1 (100.0%)	
5^th^	1	6 (66.7%)	3 (33.3%)	<0.001
	2	11 (20.4%)	44 (79.6%)	
	3	31 (88.6%)	4 (11.4%)	
	4	0 (0.0%)	3 (100.0%)	
	Association (1, 3 and 4)	3 (100.0%)	0 (0.0%)	
6^th^	1	9 (100.0%)	0 (0.0%)	<0.001
	2	9 (18.8%)	40 (81.2%)	
	3	30 (75.0%)	10 (25.0%)	
	4	0 (0.0%)	3 (100.0%)	
	Association (1, 2 and 3)	0 (0.0%)	1 (100.0%)	
	Association (1, 3 and 4)	3 (100.0%)	0 (0.0%)	
7^th^	1	7 (77.8%)	2 (22.2%)	<0.001
	2	10 (18.9%)	43 (81.1%)	
	3	31 (88.6%)	4 (11.4%)	
	4	0 (0.0%)	3 (100.0%)	
	Association (1, 2 and 3)	0 (0.0%)	1 (100.0%)	
	Association (1, 3 and 4)	3 (100.0%)	0 (0.0%)	
	Association (2 and 3)	0 (0.0%)	1 (100.0%)	
8^th^	1	7 (100.0%)	0 (0.0%)	<0.001
	2	10 (17.9%)	46 (82.1%)	
	3	31 (88.6%)	4 (11.4%)	
	4	0 (0.0%)	3 (100.0%)	
	Association (1, 2 and 3)	0 (0.0%)	1 (100.0%)	
	Association (1, 3 and 4)	3 (100.0%)	0 (0.0%)	
9^th^	1	6 (60.0%)	4 (40.0%)	<0.001
	2	9 (18.0%)	41 (82.0%)	
	3	32 (86.5%)	5 (13.5%)	
	4	1 (25.0%)	3 (75.0%)	
	Association (1, 2 and 3)	0 (0.0%)	1 (100.0%)	
	Association (1, 3 and 4)	3 (100.0%)	0 (0.0%)	
10^th^	1	7 (70.0%)	3 (30.0%)	<0.001
	2	10 (18.9%)	43 (81.1%)	
	3	31 (86.1%)	5 (13.9%)	
	4	0 (0.0%)	3 (100.0%)	
	Association (1, 3 and 4)	3 (100.0%)	0 (0.0%)	

Chi-square test with Yates's continuity correction, α=5%.

1= Alkylating agents (Cyclophosphamide, Ifosfamide, Melphalan, Dacarbazine, among others); 2= Antimetabolites (Methotrexate, 5-Fluorouracil, Cytarabine, Mercaptopurine, among others); 3= Natural products (Vincristine, Vinblastine, Etoposide, Daunorrubicin, Doxorrubicin, Interleukin-2, L-asparaginase, among others); 4=Miscelaneous (Cisplatin, Carboplatin, Procarbazine, among others)

## Discussion

Oral changes caused by oral mucositis are the most significant comorbidities after the start of chemotherapy,^8^^–^^11^ and based on the assumption that these manifestations may be different between groups of patients with solid and hematologic tumors, we conducted this study to evaluate this hypothesis, seeking to serve as a guide for decision making in oral health care for these patients.

According to previous studies, the incidence of oral complications resulting from chemotherapeutic treatments ranges from 30% to 100% in pediatric cancer patients,^7^,^20^^–^^22^ thus, these children are a high-risk group for developing oral manifestations due to the high mitosis rates in the oral mucosa.^23^

This study conducted a prospective evaluation of patients from 0 to 18 years old who were subjected to chemotherapy to treat solid or hematologic malignant tumors for a follow-up period of 10 weeks. The outcome of interest was the occurrence of changes in the components of the stomatognathic system according to the modified OAG.^19^ The main researchers in pediatric oncology have used this instrument worldwide,^23^^,^^24^ and allows the weekly monitoring of the oral health conditions in pediatric cancer patients, evaluating eight sites/functions of the stomatognathic system and classifying them according to the degree of impairment, from normal conditions to moderate and severe alterations in functions and oral mucosa.

The results of this study did not show significant differences in the changes verified in the voice, lips, tongue, and saliva (according to the OAG) between patients with solid or hematologic tumors. This finding suggests that studies on and the oral care for these patients should not be different for the two groups of patients. In other words, concerns about the prevention and monitoring of oral changes should be similar for both groups because the patterns of impairment were the same for both solid and hematologic tumors over the initial 5-week period of chemotherapy.

However, starting from 6^th^ week of chemotherapeutic treatment, significant differences were found between patients with solid and hematologic tumors in oral changes, being these alterations verified in labial mucosa, mucous membrane, gingiva and swallowing. Such difference was identified in all weeks (6^th^, 8^th^, 9^th^ and 10^th^), with moderate and severe alterations in labial mucosa, mucous membrane and gingiva being more common in the hematologic group. On the other hand, the alterations in swallowing were more significant in group of solid tumor patients in 10^th^ week of treatment. The existence of a greater impairment in patients with hematologic tumors from the 6^th^ week onwards suggests that these patients should receive a special attention from the beginning of the 2^nd^ month of antineoplastic treatment, focusing on approaches that can prevent serious impairments, especially in the labial mucosa. The concern with these patients becomes even greater because their hematologic malignancies compromise the immune system, disrupting its homeostasis.^25^^,^^26^ Therefore, this group of patients may already start chemotherapy with a level of immunosuppression, and this condition is one of the main systemic effects recorded during chemotherapy.^25^ Thus, since patients with hematologic cancers show more serious degrees of impairment than those with solid tumors in the labial mucosa, the risk of systemic compromise through the ulcerated labial mucosa is even more worrying, and we expect that the results of this study can be used to change some care practices in the oral health of patients, possibly including oral health services in the multiprofessional team that assists children and adolescents in cancer therapy.

In the 10^th^ week of evaluation, an additional concern arose because of the greater changes in swallowing among patients with solid disease. This oral ability is crucial for children and adolescents to reach the nutritional indices that support antineoplastic therapies.^27^ Although worse swallowing impairments have been assessed among patients with solid tumors, moderate degrees of impairment might progress to greater functional impairments in patients with hematologic tumors, and according to our results, moderate alterations were more frequently found in patients with hematologic tumors; therefore, the oral care should be equally focused in both groups of neoplasms.

The differences found between the patient groups evaluated in this study may have occurred due to the condition of the organism according the disease type (hematologic or not),^28^ added to immunosuppression after starting the chemotherapy treatment,^22^ which is substantially higher in patients with hematologic disease, and in addition to the type of the chemotherapy used in the treatment of the cancer. Regarding the type of chemotherapy, this study found that the antimetabolite agents – the most toxic chemotherapeutics to the stomatognathic system according to the literature –,^6^^–^^9^ were administered mainly to patients with hematologic tumors during almost all the evaluation period of this study.

Data from this study enabled the identification of the impaired sites with the highest severity, as well as the groups of neoplasms with the highest degrees of impairment, allowing for indications for early treatment to prevent the onset of systemic complications that result from infectious processes on the oral cavity.^28^

All patients followed in this study were under a permanent surveillance protocol regarding the changes of normality in the structure and function of the oral cavity, as well as being part of a protocol for the prevention and treatment of injuries that included the topical application of low power laser (calibrated to a 670 nm wavelength, 40 mW power, and 4 J/cm^2^ dose for 30 s in selected points of oral mucosa) and use of mouthwash with a multicomponent solution [nystatin (20 ml), dexamethasone (1 ml), diphenhydramine (1 ml), morphine (1 ml), lidocaine 2% (10 ml), B complex (2 ml) and saline solution 0.9% (250 ml)],^20^ in addition to oral hygiene monitoring.

The limitations of this study include the methodological difficulties related to the fact that pediatric cancer (0-19 years) is rare when compared to cancers in the other age groups, which, even using a convenience sample with the inclusion of all diagnosed patients over a 4-year period, still leads to a reduced number sample when compared to oncology studies with other age groups. Moreover, the late diagnosis of pediatric cancers in Brazil is a reality that compromises patient survival and requires cohort monitoring. However, this study evaluated patients over a long time period when compared to other studies at other international centres.^24^^,^^28^ Despite the losses, the study sample enabled a satisfactory comparison among groups, as performed in this study.

This study showed that during the first month of chemotherapy treatment (up to the 5^th^ week), the oral alterations changed equally between patients with solid and hematologic tumors, and, after the 1^st^ month (6^th^, 8^th^, 9^th^ and 10^th^ weeks), differences were observed between the groups of patients, with greater involvement by oral mucositis – especially by severe oral mucositis –, in patients with hematologic tumors. These results show the importance of the implementation of oral care in the multiprofessional team that provides care to children and adolescents in their treatments against a cancer due to the identification of oral care needs both in alterations in the oral mucosa and oral functions.

## Conclusion

This study concluded that the oral changes during the chemotherapeutic treatment occurred especially in swallowing function, in the mucous membrane, in the labial mucosa and in gingiva, and these alterations were mainly presented by patients with hematologic tumors.
